# Location, Clinical Presentation, Diagnostic Algorithm and Open vs. Arthroscopic Surgery of Knee Synovial Haemangioma: A Report of Four Cases and a Literature Review

**DOI:** 10.3389/fsurg.2021.792380

**Published:** 2021-12-07

**Authors:** José A. Hernández-Hermoso, José Moranas-Barrero, Ester García-Oltra, Fernando Collado-Saenz, Sylvia López-Marne

**Affiliations:** ^1^Department of Orthopedic Surgery and Traumatology, Hospital Universitari Germans Trias i Pujol, Badalona, Spain; ^2^Department of Surgery, Faculty of Medicine, Universitat Autònoma de Barcelona, Bellaterra, Spain; ^3^Department of Orthopedic Surgery and Traumatology, Hospital Universitari Bellvitge, L'Hospitalet de Llobregat, Spain

**Keywords:** synovial, haemangioma, knee, magnetic resonance, arthroscopy

## Abstract

**Objective:** The aim was to report 4 patients with intra-articular knee synovial haemangioma (KSH) and to perform a systematic review to describe the patient characteristics, patterns of tumor location, clinical presentation, usefulness of imaging examinations, pros and cons of arthroscopic vs. open resection, and follow-up in the literature.

**Design:** From 1996 to 2016, four patients with KSH were retrospectively reviewed. A literature search was conducted in PubMed from 2000/01 to 2020/06 using the search terms “synovial haemangioma” and “knee.” Fifty full-text articles that included a total of 92 patients were included for further discussion.

**Results:** Four adults (20–40 years) were diagnosed with KSH. Three lesions located in the suprapatellar pouch, two eroding the patella and one the supratrochlear bone, and one in the posterior compartment. Persistent anterior knee pain was the main complain. MRI revealed a benign tumor mass in all cases except one. Open excisional biopsy and regional synovectomy were performed in three patients, and by arthroscopy of the posterior compartment in the fourth. Histological type was arteriovenous in three cases and capillary in one. A pain-free knee without recurrence was achieve in all cases except one, which was successfully reoperated. Average follow-up time was 3.5 years. A literature review showed that KSH appears most frequently in children and teenagers (64.6%) and does not differ by gender. The suprapatellar and patella-femoral joint compartment was the most frequent location (47.9%). The bony tissue of the knee was rarely affected (13.5%). Pain, swelling and haemarthrosis were frequently reported (88.2, 66.7, and 47.1%). MRI was the most commonly used imaging test (98%). Treatment consisted of regional synovectomy by open surgery or arthroscopy in 66.7 and 15.6% of cases, respectively.

**Conclusions:** KSH should be considered in the differential diagnosis of adult patients with chronic low-intensity knee pain. MRI is the most useful exam because it establishes the location, extent and benign characteristics of the tumor. Definitive diagnosis requires histological examination. We believe excisional biopsy and regional synovectomy by arthroscopy should be the treatments of choice for intra-articular tumors, but we recommend open surgery when the lesion extends to the tendons, muscle or bone.

## Introduction

Synovial haemangioma (SH) is a rare benign tumor accounting for 0.07% of all soft tissue tumors and 0.78% of all excised haemangiomas (1). While this type of lesion is usually monoarticular (2), more than one mass can occur in different locations within the joint (3). SH has been reported in many joints but is most common in the knee (4).

Knee synovial haemangioma (KSH) can present as diffuse or localized intra-articular, juxta-articular or intermediate (intra- and juxta-articular) synovial tissue masses (2, 5–7). Extra-synovial and intra-synovial locations have been described for joint tumors (8), with intra-synovial locations being more likely to affect cartilage (8). It can be a part of an angiodysplastic lesion of the extremities (Klippel-Trénaunay syndrome) with diffuse (9, 10) or localized (11) involvement of the joint.

Most are located in the anterior compartment of the knee (7, 12). Only eight localized cases have been reported in the posterior compartment [one intermediate (5), three juxta-articular (1, 12) and four intra-articular (13–17)], one of which was reported twice (14, 15). Most reports are single case studies (1–3, 11, 13–50), while case series are less common (4–10, 12, 51–57).

The clinical presentation is non-specific, with symptom onset usually occurring in childhood and young adulthood, although it has been described at later ages, probably due to delayed diagnosis. Various imaging modalities have been used, but all are non-specific, and there is no clear diagnostic algorithm (2, 4, 9, 13, 18, 25, 28–30, 32, 33, 38, 45, 48, 50, 55, 57, 58).

Once a benign synovial lesion is suspected, it is unsettled if a biopsy is needed or if excisional biopsy and regional synovectomy with histological examination can confirm the diagnosis and simultaneously accomplish definitive treatment. Whether open (2–5, 8–10, 16, 21, 37, 38, 57) surgery is preferable to arthroscopic (6, 38, 39) surgery remains controversial, especially in localized intra-articular lesions. There is no clear information on the recurrence rate after surgical treatment.

Further investigation is warranted, particularly because the existing information is scattered and based mostly on isolated clinical cases. An understanding of the differences in patient characteristics and clinical presentation, along with knowledge of the real value of imaging examinations and follow-up results after arthroscopic or open surgery, is important for making informed decisions on the need for invasive imaging examinations and on the differential effectiveness of open vs. arthroscopic treatment in light of the evidence.

The aim of the study was to describe four patients with intra-articular KSH, two with the previously undescribed phenomenon of patellar bone erosion and one with an unusual tumor location in the posterior compartment, and to perform a systematic review of PubMed publications on KSH from 2000 to 2020 to describe the characteristics of the patients (age, gender, knee side, previous trauma), the patterns of tumor location, the non-specific clinical presentation, the usefulness of imaging examinations, the pros and cons of arthroscopic vs. open resection, and the follow-up time and recurrence after treatment.

## Materials and Methods

### Clinical Cases

Based on hospital records, we identified four patients with KSH who were seen between 1996 and 2016. Demographic data, symptoms, imaging tests, treatment and follow-up were retrospectively reviewed. All patients underwent surgical treatment by one surgeon (JHH). Written informed consent was obtained from the patients in all cases, and the retrospective study was approved by the hospital ethical committee (approval number PI-20-196). The procedures followed were in accordance with the ethical standards of the responsible committee on human experimentation (institutional and national) and with the Helsinki Declaration of 1975, as revised in 2000.

### Literature Review

A PubMed literature search was performed for full text publications between 2000/01 and 2020/06; these dates were selected because MRI was widely available in this period. The search terms were as follows: (synovial haemangioma) AND (Knee) AND (2000/01:2020/06). Selection was restricted to single case reports or case series of KSH that described the diagnosis and at least four items in our study objectives. Only original reports or studies written in English, Spanish or French and available in full-text form were included.

Each original manuscript was scrutinized, and search results on the number of cases described, the age and gender of the patients, the side of the affected knee, the tumor location, the history of previous trauma, the symptoms and signs, the presence of cutaneous haemangioma, the delay in diagnosis, the image examinations performed, the presence of bone involvement, the type of treatment, the histological type and the follow-up time were screened and collected in a database by two reviewers independently (JHH, JMB) to ensure accuracy and then stored on a Microsoft Excel spread sheet. Any discrepancy in the data collected was reviewed by both reviewers until an agreement was obtained. The review was limited to reporting variables that were obtained in each publication; when a variable was omitted, it was registered as not reported. The results are presented as the number of cases and the percentage with respect to the total number of cases, except for symptoms and signs and image examinations, which are presented as the number of manuscripts where they are reported and the percentage with respect to the total number of manuscripts reviewed. Age, delay in diagnosis and follow-up time are presented as the mean ± standard deviation of data available, and the percentage of cases not reporting those data was also specified. In order to avoid multiple reports of the same study, studies with the same authors were double-checked, and comparisons of case descriptions and treatments were performed. Neither of the review authors was blinded to the journal titles or the study authors or institutions.

## Results

Results tabulated by age, gender, affected knee side, tumor location, history of previous trauma, clinical symptoms and signs, presence of cutaneous haemangioma, time in diagnosis delay, imaging test used, bone involvement, type of treatment, histology, follow-up time and recurrence are shown in Table 1 for the clinical cases and Table 2 for the literature review.

**Table 1:** Clinical case demographics, tumor location, clinic, image examinations, treatment, histology, and follow-up.

	**Age**	**Gender**	**Side**	**Location**	**Trau**	**Symptoms/Signs**	**C H**	**DeD**	**Image Examinations**	**B A**	**Treat**	**Histology**	**F-up**	**Rec**
Case 1	20	M	R	SP-Q	No	P-H	Nr	5 y	XR-Sc-MRI	Yes	OS	AV	4 y	Yes
Case 2	22	M	R	SP-Q	No	P	Nr	6 y	XR-MRIN-MRI	Yes	AN-OS	AV	5 y	No
Case 3	31	M	R	SP	No	P-PA-S-T	Nr	8 y	XR-MRI	Yes	OS	Cp	4 y	No
Case 4	40	M	R	PC	No	P-PA	Nr	10 y	XR-MRI	No	AS	AV	1 y	No

*Trau, previous trauma; CH, Cutaneous haemangioma; DeD, Delay in diagnosis; BA, Bone Affected; Treat, Type of treatment; F-up, Follow-up; Rec, Recurrence; y, years; F, Female; M, Male; R, Right; L, Left; SP, Suprapatellar; PC, Posterior compartment; Q, Quadriceps; P, Pain; PA, Pain with Activity, Tenderness H, Hemarthrosis; XR, radiography; Sc, Scintigraphy; MRI, Magnetic resonance image; MRIN, MRI negative; OS, Open surgery; C, Curettage; AN, Arthroscopy Negative; AS, Arthroscopic Surgery; Cp, Capillary; AV, Arteriovenous; Nr, not reported*.

**Table 2:** Knee synovial haemangioma literature review summary 2000–2020.

	***N*°**	**Age**	**Gender**	**Side**	**Location**	**Trau**	**Symptoms/Signs**	**C H**	**DeD**	**Image Examinations**	**B A**	**Treat**	**Histology**	**F-up**
Our series 2020	4	20-22-31-40 y	4 M	4 R	SP (3) Q (1)- PC	No	P-PA-S-T-H	Nr	7.2 y	XR-Sc-MRIN-MRI	Yes (3)	OS (3)-AS	AV (3)-Cp	3.5 y (1 R)
Muramatsu et al. (12)	10	12.4 y (4 m−17 y)	5 F-5 M	Nr	PFL (3)-PFM (2)-IP (1)-PC (2)-D (2)	yes	P-H-LRM	No	2.5 y	XRP (1)-MRI	No	AB-OS	C (2)-Cp (6)-CCp (2)	1 y
Tohma et al. (3)	1	41 y	M	R	MC	Nr	P-S- PTM-LRM	Nr	30 y	XRB-MRI-MRA	Yes	OS	AV	4 y
Goki-Kemei et al. (25)	1	38 y	F	R	MC	No	PA-S-T-E-LRM	Nr	Nr	MRI	No	AB-OS	SH	3 m
Bawa et al. (21)	1	25 y	F	R	PFL	No	P-S-E-PTMF	No	20 y	XR- MRI	No	AS	C	Nr
Beltrame et al. (23)	1	7 y	F	R	IP	Nr	P-S	Nr	Nr	MRGd	No	Nr	Nr	Nr
Begly et al. (22)	1	39 y	M	R	IN	Yes	P-L-LRM-T-E-H	No	1 w	XR-MRI	No	AS	SH	Nr
Wen et al. (46)	1	14 m	F	R	IPL-IN	No	RW-Lu-PTM-H	Nr	3 w	XRS-US-MRGd-	No	OS	C	Nr
Derzsi et al. (1)	1	13 y	M	L	PC	Yes	S-PTM-A-LRM	Nr	2 y	XR-MRI	No	OS	AV	21 m
Masquefa et al. (15)	1	37 y	F	Nr	PC	Nr	Nr	Nr	Nr	MRI	No	AS	SH	22 m
Dunet et al. (14)						Yes	P	No	9 m	Rx-CT-MRGd	No		C	5 y
Lopez-Oliva et al. (54)	4	22 y (6–43 y)	2 F-2 M	Nr	IN-MC-SP-IP	Nr	P-S-A-LRM-H	Nr	3 y	XRP (1)-XRO (3)-MRI	No	OS (3)-Ob	Nr (4)	6.8 y
Arslan et al. (19)	1	23 y	M	L	SP-VMO	Nr	P-S-T	Nr	1.5 y	XR-US-CT-MRGd	No	AB-Nr	SH	Nr
Guler et al. (27)	1	19 y	M	Nr	SP	Nr	P-S-T	Nr	Nr	XR-MRGd	No	Nr	Nr	Nr
Andrea Parra et al. (50)	1	2 y	M	R	SP-VL	Nr	P-S-M	Nr	18 m	XRS-ED-MRI	No	OS	VVM	Nr
Mrani Alaoui et al. (34)	1	7 y	M	L	D	Yes	P-S-L-LRM-A	No	12 m	XR-MRGd	Yes	OS	Cp	36 m (R)
De Gori et al. (26)	1	67 y	M	L	D	Nr	P-S-Cl-Cr-T-LRM	Nr	15 y	XRO-MRI	No	TKA	SH	2 y
Kim et al. (30)	1	7 y	M	R	IP	Nr	S-H	Nr	1 y	MRI-RBCSc-SPECT/CT	No	OS	V	Nr
Maeyama et al. (33)	1	15 y	F	R	MC	No	PA-S-A-LRM	Nr	7 y	XRBO-Ar-CT-MRI	Yes	AS	SH	5 y (SP)
Tahmasebi et al. (42)	1	45 y	F	R	PFM	No	PA-S-PTM-H	Nr	Nr	XR-MRI	No	AS	C	1 y
Wong et al. (48)	1	5 y	M	R	IPM	No	P-S	Nr	2 y	MRI	No	OS	SH	32 m
Dalmonte et al. (8)	14	5 y (2.5–13 y)	8 M-6 F	Nr	SIE-IIE-II (4 in two or more)	Nr	P-S-LRM-H	Yes (7)	26 m	XRS (6)-XRP (2)-US-MRI	No	AB-OS	IAVM (14)	46 m (2 R) (3 SP)
Hospach et al. (28)	1	15 y	M	R	SP-Q-B	Nr	P-S-LRM	Nr	6 y	XR-US-ED-MRIN-MRGd	No	AN-AS	C	Nr
Lin et al. (32)	1	11 y	M	L	SP-IN	No	P-S-LRM-H	Yes	1 y	XR-MDCTA	No	Cor-Bblo	Nr	2 m
Sasho et al. (59)	2	37–60 y	F-M	R (2)	IP-SP	Nr	P-H	Nr	19 y	XR-MRGd	No	AN-AS	C (2)	2 y
Vakil-Adil et al. (44)	1	12 y	M	L	SP-VMO	Nr	P-S-PTM flexion	No	3 y	XR-MRI	No	OS	C	Nr
Watanabe et al. (45)	1	3 y	F	L	IP	No	P-S-Li-E	No	6 m	XRB-Sc-MRGd	Yes	AB-Ob	C	6 m (R) (SP)
Holzapfel et al. (2)	1	29 y	M	L	MC	No	PA cross leg	No	3 m	XRB-Sc-CT-MRGd	Yes	OS-C-BG	C	Nr
Ares-Rodriguez et al. (18)	1	10 m	M	L	IPM	Yes	P-Ed-Li-H	Nr	24 h	Ar-MRI	No	OS	AV	4 y
Rajni et al. (37)	1	10 y	F	R		No	D	Nr	4 y	XRS-MRI	No	AB-OS	CCp	Nr
Tzurbakis et al. (43)	1	34 y	M	R	IN	Yes	P-S-L-A	Nr	5 y	XR-MRI	No	AS	Cp	4 y
Barakat et al. (60)	1	24 y	F	L	PFM	No	P-H	Nr	15 y	ED-MRI	No	AN-AS	C	12 m
Carrol and Higgs (24)	1	10 y	M	R	SP	No	S-W-LRM-H	Nr	8 y	XRC-US-MRI	Nr	Nr	Nr	Nr
Sanghi et al. (38)	1	7 y	F	L	SP	Yes	RW-LRM-PTM	Nr	3 y	XR-ED-MRGd	No	AS	C	Nr
Silva et al. (40)	1	20 y	M	R	SP	No	P-S	Nr	6 m	XR-MRI	No	AS	SH	2 y
Yercan et al. (55)	3	11 y-21 y-22 y	3 F	2 R-l L	SP (3) VMO (1)	No	P-S-B-T-E-A-PTM-LRM-H	Nr	6 y	XRS-CT-MRI	Yes (1)	AB-OS-C	CCp (1)-Nr (2)	38 m
Zarza Pérez et al. (11)	1	37 y	F	R	IP	Nr	P-S-B-A-H	SA	5 y	XR-MRI	No	AS	SH	1 y
De Filippo et al. (52)	4	19–24 y	3 F-1 M	Nr	IP (1)-Nr (3)	Nr	P-S-E	Nr	6 y	XR-MRGd	No	AB-Nr	C (4)	Nr
Winzenberg et al. (47)	1	34 y	F	R	IN	Yes	P-Li-GW-L	Nr	20 y	XR-MRIN	No	AS	Cp	18 m
Coulier (13)	1	19 y	F	Nr	PC	Yes	P	Nr	3 y	XR-CTArt-CTA-MRI	No	Ob	Nr	Nr
Okahashi et al. (35)	1	24 y	F	R	PFM	No	P-S-T-A	NR	20 y	XR-MRI	No	AB-OS	Cp	2 y
Ramseier et al. (10)	4	9-5-7-10 y	2 F-2 M	3 L-1 R	SPM-SPL-IPM-IP	Yes (1)	PW-LL-S-PTM	KT (2)	3.9 y	XRO-XRP (1)-MRI	No	OS	SH (3)-C	2.7 y (1 R) (4 SP)
Yilmaz et al. (49)	1	25 y	M	R	SP-Q	No	P-S-E-A	Yes	10 y	XR-V-MRI	No	AB-Ob	VVM	1 y
Akgün et al. (6)	4	19-22-13-35 y	2 F-2 M	4 R	2 D-2 SP	Yes	P-S-B-E-H	Yes (2)	4.5 y	XRO (1)-MRI	Yes (1)	AB-Ob (2) OS-AS	SH (3)-C	2.6 y (2 SP)
Bonaga et al. (9)	4	4-18-20-49 y	1 F-3 M	3 L-1 R	D (4)	Nr	P-SL-LRM-H	SA (2)-KT (2)	Nr	XRO (1)-V-Ar-MRGd	No	AB-OS	AV (4)	10 y (1 SP)
Del Notaro and Hug (17)	1	30 y	F	L	PC	Yes	PA-Li-E-LRM-H	Nr	1 y	XR-MRI	No	ASF-OS	C	Nr
Neel et al. (16)	1	10 y	M	L	PC	Yes	P-L-E	No	2 m	XR-MRGd	Yes	OS	SH	Nr
Suh et al. (41)	1	59 y	M	L	SPL	Nr	P-S-PTM-LRM	Nr	40 y	XR-MRI	No	AB-OS	Cp	6 m
Abe et al. (5)	3	5-12-16 y	2 F-1 M	2 R-1 L	SPL VL-SP-PF Q Gn	Nr	P-S-A-LRM-H	Nr	8.3 y	XRP (2)-XRO (2)-MRI	No	AB-OS	C (2)-Cp	7 y
Lassoued et al. (31)	1	25 y	M	R	D	No	P-H	No	20 y	XRO-XRB-MRI	Yes	AN-OS	C	4 y
Silit et al. (39)	1	39 y	M	R	MC	Nr	P-S-H	No	10 m	XR-MRGd	Yes	ASI	CCp	1 y
Aynaci et al. (20)	1	15 y	F	L	IPL	No	P-T-A	Nr	6 m	XR-MRI	No	AS	VVM	1 y
Total	96	Cases X ± SD 21.7 ± 14.7 y 0–10 y 34.4% 11–20 y 30.2% 21–30 y 16.7% 31–40 y 11.5% 41–50 y 4.2% >51 y 3.1%	Cases F 47 (49%) M 49 (51%)	Cases R 38 (39.6%) L 23 (24.0%) Nr 35 (36.5%)	Cases SP-PF 46 (47.9%) IP 14 (14.6%) IN 4 (4.2%) MC 7 (7.3%) PC 9 (9.4%) D 13 (13.5%) Nr 3 (3.1%)	Cases Yes 13 (13.5%) No 38 (39.6%) Nr 45 (46.9%)	Manuscript P 45 (8.2%) PA 17 (17.6%) CL 1 (2%) Cr 1 (2%) RW 1 (2%) L 3 (5.9%) Li 7 (13.7%) Lu 1 (2%) LL 1 (2%) SL 1 (2%) B 3 (5.9%) GW 1 (2%) A 11 (21.6%) S 34 (66.7%) T 10 (19.6%) W 1 (2%) E 9 (17.6%) PTM 7 (13.7%) LRM 17 (33.3%) H 24 (47.1%)	Cases Yes 19 (19.8%) No 28 (29.2%) Nr 49 (51.0%)	Cases X ± SD 6.8 ± 8.6 y	Manuscript XR 47 (86.3%) MRI 50 (98.0%) MRGd 15 (29.4%) Ar 4 (7.8%) V 2 (3.9%) US 5 (9.8%) ED 4 (7.8%) CT 3 (5.9%) CTArt 1 (2.0%) CTA 1 (2%) MDCTA 1 (2%) Sc 3 (5.9%) RBCSc 1 (2%) SPECT/CT 1 (2%)	Cases Yes 13 (13.5%) No 82 (85.4%) Nr 1 (1.0%)	Cases OS 64 (66.7%) AS 15 (15.6%) TKA 1 (1.0%) Ob 8 (8.3%) Nr 8 (8.3%)	Cases C 27 (28.1%) Cp 13 (13.5%) CCp 5 (5.2%) AV 7 (7.3%) VVM 17(17.7%) SH 19 (19.8%) Nr 8 (8.3%)	Cases X±SD 2.7+-2.2y Nr (36%) R: 3.1% SP: 11.4%

*Trau, previous trauma; CH, Cutaneous haemangioma; DeD, Delay in diagnosis; BA, Bone affected; Treat, Type of treatment; F-up, Follow-up; m, Months; y, Years; w, Weeks; R, Recurrence; SP, Symptoms persisted; F, Female; M, Male; R, Right; L, Left; PFL, Patello-femoral lateral; PFM, Patello-femoral medial; IP, Infrapatellar (Hoffa fat pad); IPL, Infrapatellar Lateral; SP, Suprapatellar; SPL, Suprapatellar Lateral; MC, Medial Compartment; IN, Intercondylar Notch; PC, Posterior compartment; D, Diffuse; VMO, Vastus Medialis Obliquus; VL, Vastus Lateralis; Q, Quadriceps; Gn, Gastrocnemius; P, Pain; PA, Pain with Activity; Cl Claudication; Cr, Crepitus; RW, Patient Refused Weightbearing; L, Locking; Li, Limping; Lu, Lump; LL, Long Leg; SL, Short Leg; B, Buckling; GW, Giving Way; S, Swollen; T, Tenderness; W, Warmth; E, Effusion; A, Atrophy; PTM, Palpable tumor mass; PTMF, Palpable Tumor Mass more pronounced in Flexion; LRM, Limited Range of motion; H, Haemarthrosis; SA, Systemic Angiomatosis; KT, Klippel-Trénaunay; XR, radiography; XRS, XR Soft Tissue Mass; XRO, XR Osteoarthritis; XRB, XR Bone Involvement; XRP, XR Phleboliths; Ar, Arteriography; V, Venography; US, Ultrasound; ED, Echo Doppler; CT, Computed Tomography; CTArt, CT Arthrography; CTA, CT Angiography; MDCTA, Multi Detector Computed Tomography Angiography; Sc, Scintigraphy; RBCSc, Red blood cell scintigraphy; SPECT/CT, single-positron emission computed tomography-computed tomography; MRI, Magnetic resonance image; MRIN; MRI negative; MRA, MRI angiography; MRGd, MRI gadolinium enhancement; OS, Open surgery; C, Curettage; BG, Bone Graft; AN, Arthroscopy Negative; AB, Arthroscopic biopsy; AS, Arthroscopic Surgery; ASF, AS Failed. ASI, AS Incomplete; TKA, Total Knee Arthroplasty; Cor, Corticosteroid; Bblo, Beta blockers; C, Cavernous; Cp, Capillary; AV, Arteriovenous; CCp, Cavernous and capillary; SH, Synovial haemangioma; VVM, Venous Vascular Malformation; IAVN, Intraarticular Vascular Malformation; Nr, not reported; X ± SD: media ± standard deviation*.

### Clinical Cases

#### Case 1

A 20-year-old male suffering from sudden intense pain in the right knee with swelling presented to the emergency room. He had had occasional pain for 5 years, but never at that intensity. Palpation of the external aspect of the patella was painful. There was no patellar instability; meniscal, ligamentous and neurovascular examinations were normal.

**Figure 1:**
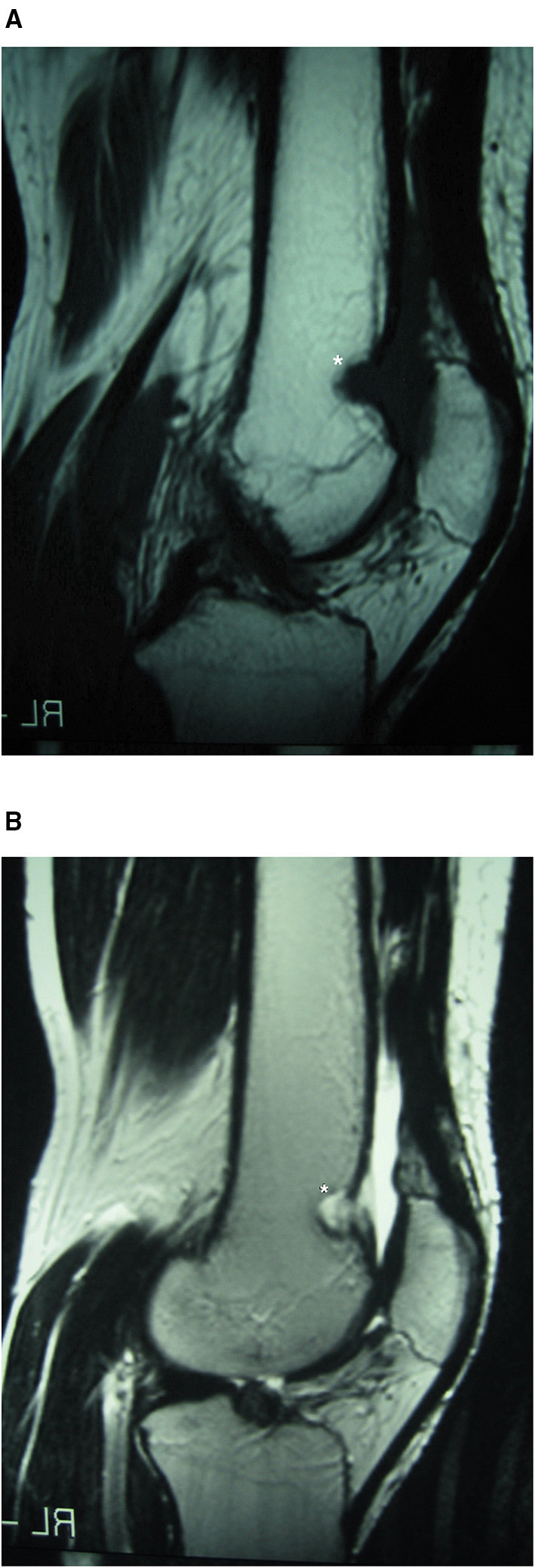
Case 1: suprapatellar SH MRI. Conventional sagittal T1 **(A)** and T2 **(B)** MRI showing synovial haemangioma in the suprapatellar and supratrochlear synovial lining extending medially and eroding the supratrochlear cortical bone, with features of hyperintensity on T2-weighted imaging and hypo-/isointensity to muscles on T1. *Location of the Synovial Haemangioma tumor in the knee.

Plain radiographs were normal. Joint aspiration revealed a haemarthrosis. Anterior knee pain syndrome due to patella-femoral cartilage damage or synovial derangement was suspected. Bone scintigraphy with 99mTc revealed an increase in uptake in the anterior portion of the knee. MR revealed a suprapatellar and supratrochlear synovial tumor extending medially and eroding the supratrochlear cortical bone; the tumor was characterized by hyperintensity on T2-weighted images and hypo-/isointensity to muscles on T1 sequences (Figures 1A,B).

**Figure 2:**
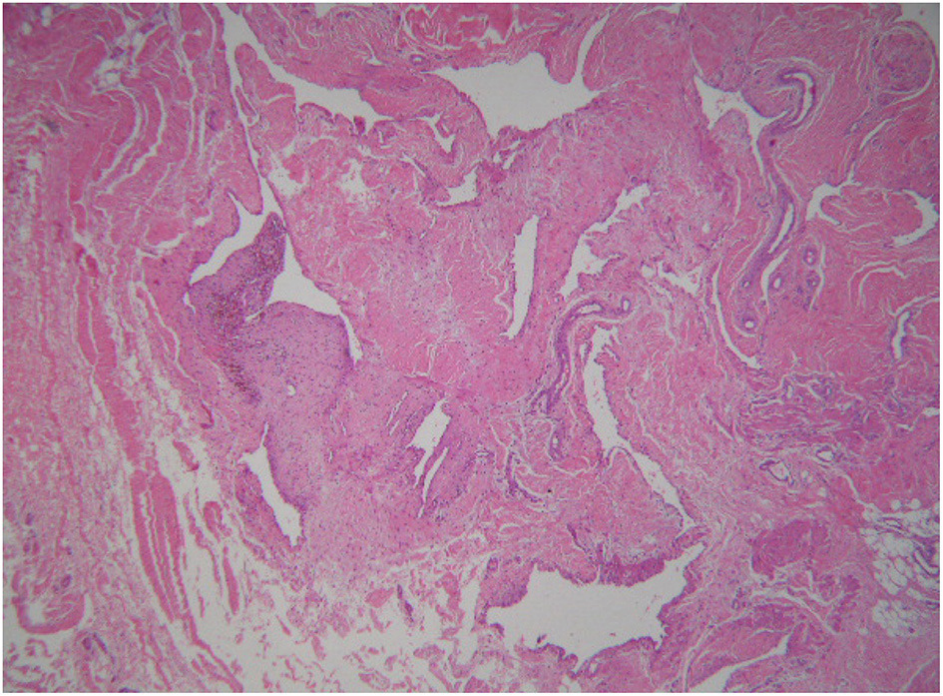
Case 1: histology. Histological findings showing proliferation of arterial and venous vessels in a fibrous stromal tissue type (haematoxylin and eosin stain, 40×). The histological diagnosis was arteriovenous synovial haemangioma.

A non-specific benign synovial knee tumor, either SH or localized pigmented villonodular synovitis (LPVNS), was suspected. An open excisional biopsy, partial synovectomy and curettage of bone were performed. Histological examination reported proliferation of arterial and venous vessels in a fibrous stroma, consistent with the diagnosis of arteriovenous-type SH (Figure 2).

**Figure 3:**
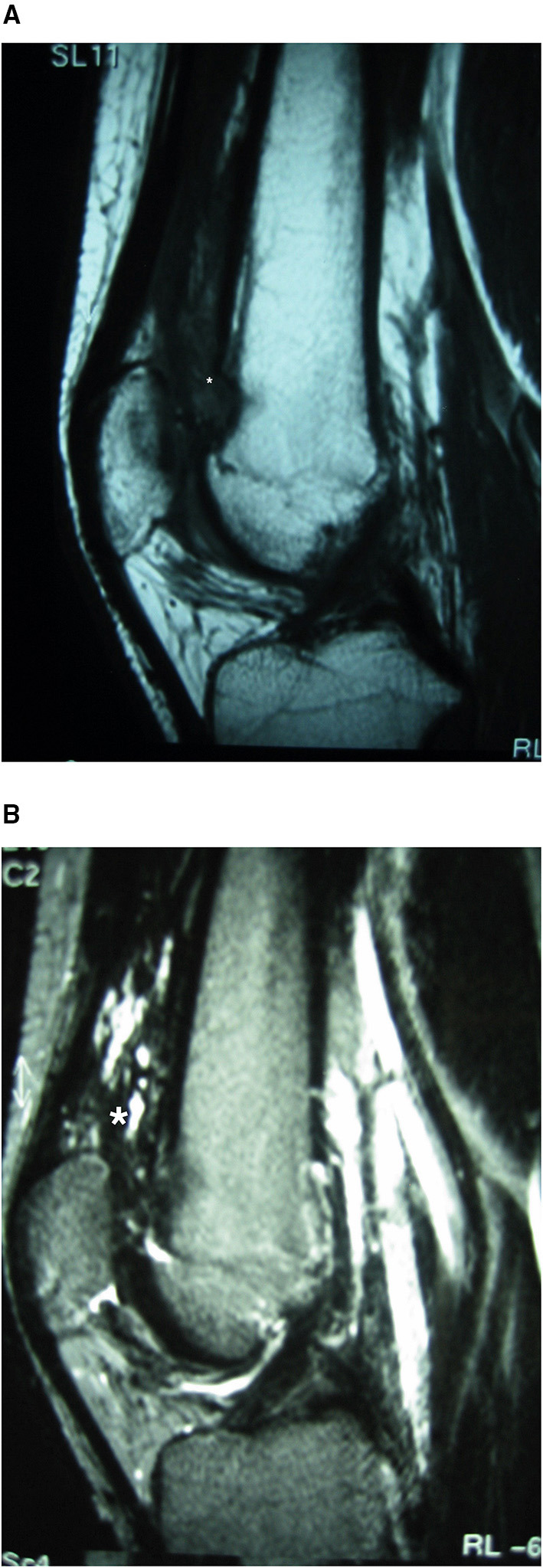
Case 1: suprapatellar MRI tumor recurrence. MRI showing recurrence of the lesion. A heterogeneous hyperintense lesion is observed on sagittal T2-weighted imaging **(B)**; it contains fibrous septa and is hypo-/isointense to muscles on the T1 sequence **(A)**, with diffuse involvement of the sub-quadriceps synovial sac and tendon. *Location of the Synovial Haemangioma tumor in the knee.

Recurrence of the tumor was observed on MRI follow-up 5 years later. MRI revealed diffuse involvement of the sub-quadriceps synovial lining and tendon (Figures 3A,B). An open wide regional synovectomy of the sub-quadriceps synovia and tendon was performed. Two months postoperatively, the patient was engaging normally in activities of daily living, and at 5 months, he resumed his amateur sport routine. Four years after the second surgery, the patient remained asymptomatic with no recurrence on follow-up MRI.

#### Case 2

A 22-year-old male was referred to our hospital for persistent anterior right knee pain lasting 6 years. He had moderate pain that did not limit activities of daily living but prevented him from practicing sports. He had no history of prior trauma, although he had experienced minor impacts during sports. On physical examination, he had lower limb varus alignment, no muscle atrophy, no effusion, normal range of motion, a stable knee, and normal meniscal and neurovascular examinations.

Radiographic images were normal. MRI was reportedly normal. Due to the persistence of symptoms despite analgesics and muscle strength exercises, diagnostic arthroscopy was performed. We detected no evidence of joint damage. Histological and microbiological tests of the synovial lining from various areas were normal.

**Figure 4:**
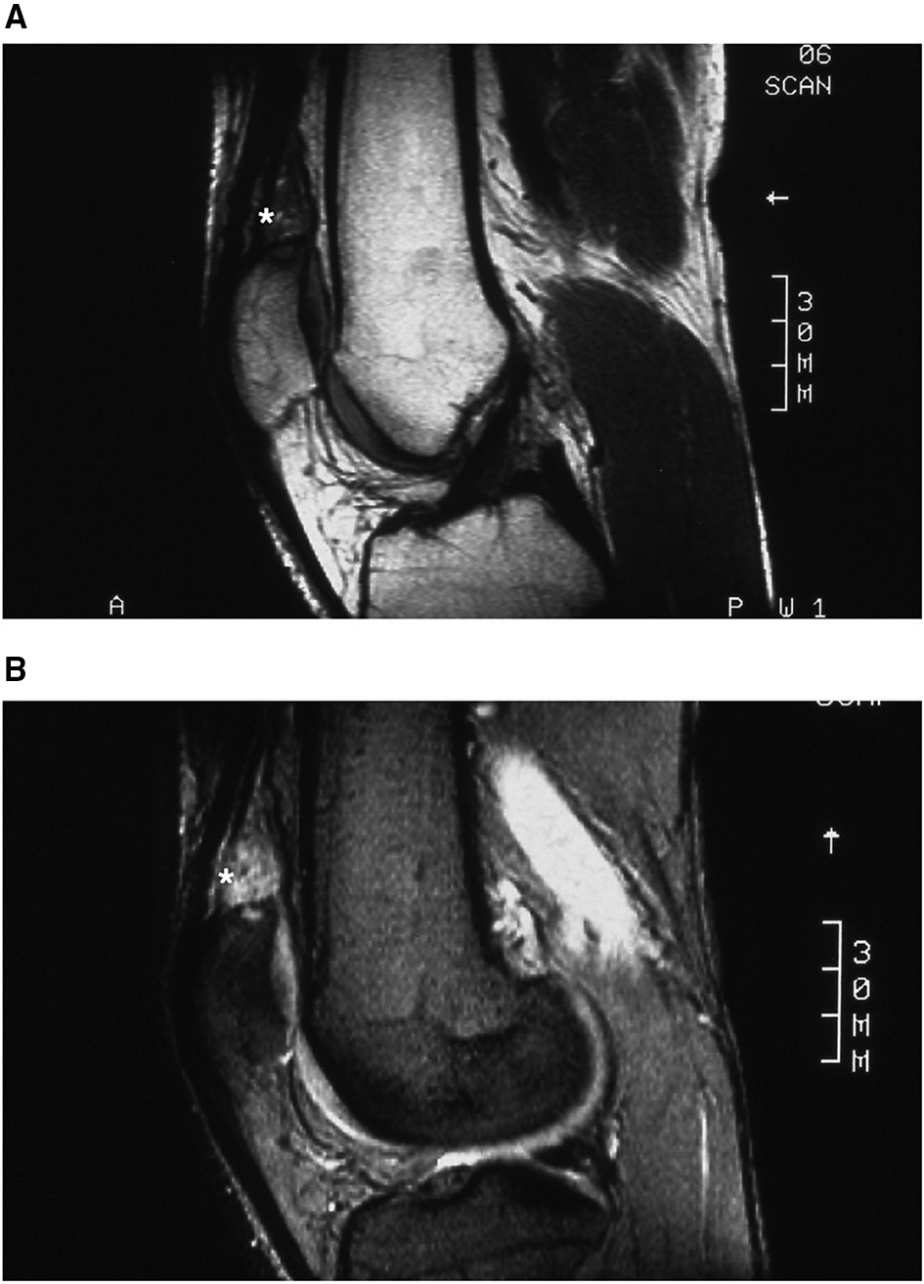
Case 2: suprapatellar SH MRI. Isointense T1 **(A)** and hyperintense T2 **(B)** sagittal MR images showing a synovial tumor just over the proximal pole of the patella with intra-osseous invasion of the upper pole of the patella. *Location of the Synovial Haemangioma tumor in the knee.

However, owing to worsening symptoms 2 years later, another MRI was performed, revealing a tumor in the synovial lining just over the proximal pole of the patella; the tumor was isointense on T1 and hyperintense on T2, with intra-osseous invasion of the upper pole of the patella (Figures 4A,B), suggestive of SH.

Upon open surgery, the synovial lining above the upper pole of the patella was swollen and bluish brown, with poorly defined margins, and there was invasion of the quadriceps tendon and the superior pole of the patella. We resected the tumor and half of the quadriceps tendon to healthy margins and curetted the superior patellar bone lesions back to normal trabecular bone. Histological examination revealed blood vessels with multiple arterial and venous vessels in the stroma, confirming the diagnosis of arteriovenous SH.

At the 6-week follow-up, the patient had a stiff knee with a range of motion of 0–30°. At 10 weeks, we performed arthroscopic arthrolysis and obtained new biopsy samples. Histological examination showed no evidence of a tumor. At the 5-year follow-up, the patient had a pain-free knee and a normal range of motion. A repeat MRI showed no evidence of recurrence.

#### Case 3

A 31-year-old man presented with an 8-year history of diffuse right knee pain, which persisted at rest and was aggravated with activity. The pain limited his participation in sports but not his activities of daily living. On physical examination, the knee was well-aligned but had mild swelling and tenderness adjacent to the medial patellar facet. There was no muscle atrophy or warmth. The range of motion and stability were normal, as were the meniscal and neurovascular examinations.

**Figure 5:**
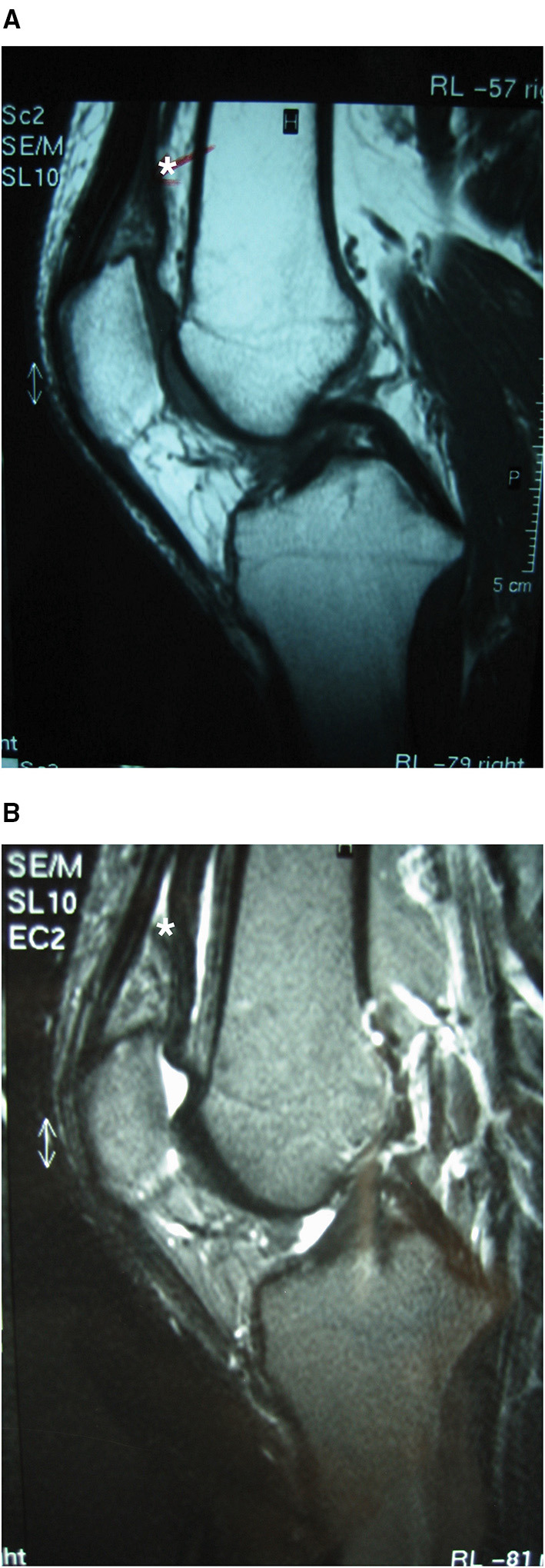
Case 3: suprapatellar SH MRI. MR sagittal images showing an intra-articular synovial mass located superior and medial to the patella that eroded the superior patellar cortical bone; the mass was heterogeneously hypointense on T1-weighted imaging **(A)** and isointense on T2-weighted imaging **(B)**. *Location of the Synovial Haemangioma tumor in the knee.

Plain radiographs were normal. On aspiration, the joint fluid was clear, and the cell count was normal with no microcrystals. We suspected anterior knee pain syndrome due to patella-femoral osteoarthritis. MR revealed an intra-articular synovial mass located superior and medial to the patella that had eroded into the superior patellar cortex. Heterogeneous lower signal intensity on T1-weighted imaging and isointensity on T2-weighted imaging suggested LPVNS (Figures 5A,B).

**Figure 6:**
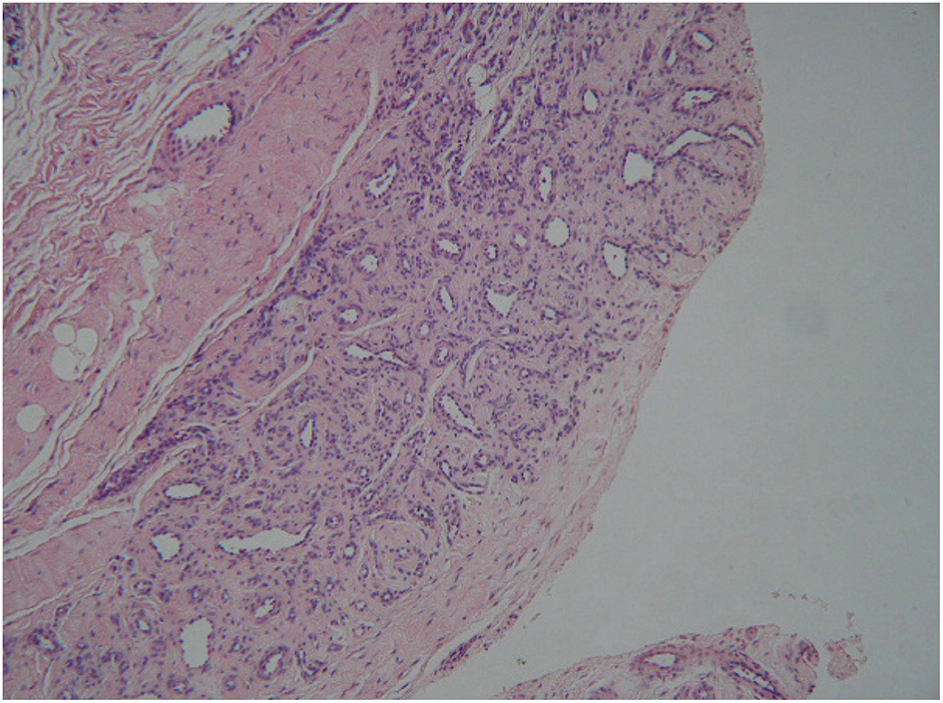
Case 3: histology. Histological examination showing synovial proliferation and capillaries below, suggesting a capillary-type synovial haemangioma (haematoxylin and eosin stain, 40×).

Open excisional biopsy and partial synovectomy were performed. Two small intra-osseous cystic lesions in the medial and superior poles of the patella were curetted until healthy trabecular bone was observed. Histological exam showed synovial proliferation with subjacent capillaries, consistent with capillary-type SH (Figure 6).

The patient had an uneventful postoperative recovery, with restoration of full range of motion and good muscle strength. He resumed amateur sports. Four years after surgery, he had a normal pain-free knee, and there was no evidence of tumor recurrence on MR.

#### Case 4

A 40-year-old male presented with a 10-year history of progressive anterior and posterior right knee pain with insidious onset. He had no history of trauma. Pain occurred when he performed maximum knee flexion or intense cycling but not during activities of daily life.

On clinical examination, both lower limbs were aligned in varus. There was no muscular atrophy, swelling, tenderness or joint effusion. The range of motion of the knee was normal, but the patient reported discomfort at maximum flexion. The knee was stable, and the meniscal and neurovascular examinations were normal.

**Figure 7:**
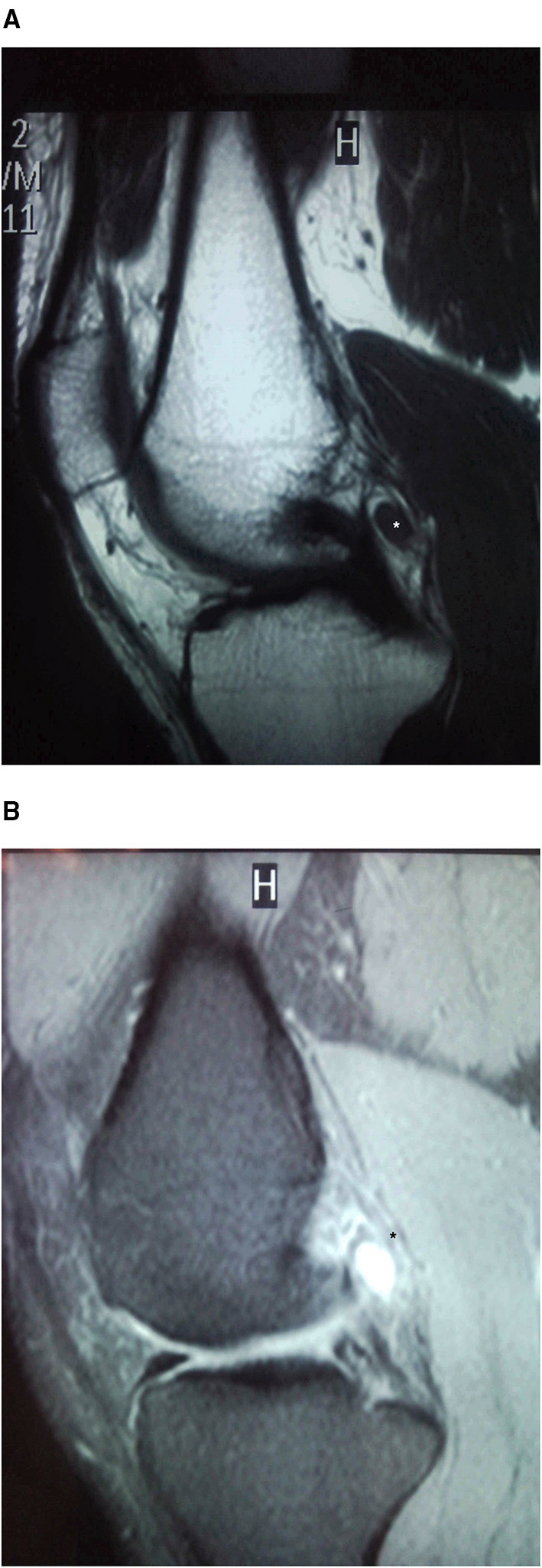
Case 4: posterior compartment SH MRI. Sagittal MRI sequences showing a well-defined intra-articular synovial mass in the posterior compartment of the knee, behind the PCL. On T1-weighted **(A)** sequences, the mass was isointense to the surrounding muscle with peripheral heterogeneous areas; the mass was hyperintense on T2-weighted images **(B)**. *Location of the Synovial Haemangioma tumor in the knee.

Plain radiographs were normal. MRI showed a well-defined intra-articular synovial mass in the posterior compartment of the knee, behind the PCL. On T1-weighted sequences, the mass was isointense to the surrounding muscle signal with peripheral heterogeneous areas, and on T2-weighted imaging, it was hyperintense (Figures 7A,B). A benign synovial tumor was suspected, with the possibility of LPVNS or SH.

**Figure 8:**
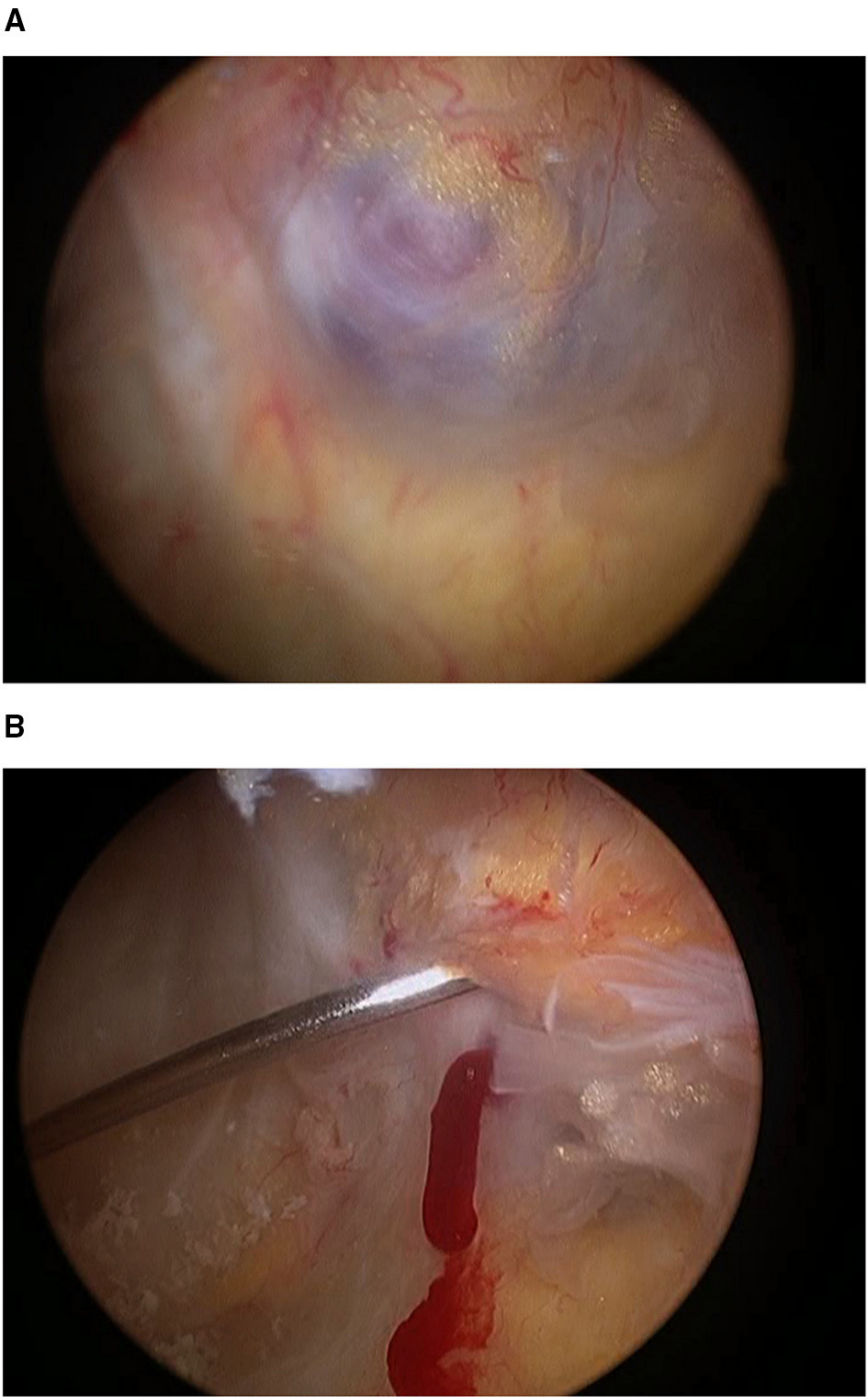
Case 4 posterior compartment SH arthroscopic view and puncture. **(A)**. Arthroscopic view of the synovial haemangioma via the anterolateral portal, showing a pale bluish vascular lesion in the synovium lining the posterior capsule, just behind the PCL (1 × 0.5 × 0.5 cm). **(B)** Puncture of the synovial tumor produced acute bleeding that differentiated it from other tumors, such as LPVNS.

The patient opted for observation and regular follow-up. Four years later, his pain had increased and was more frequently limiting his activities of daily living. Repeat MRI showed features similar to what had been observed previously, but the patient elected to proceed with surgery. On arthroscopy, we observed a 1 × 0.5 × 0.5 cm pale bluish vascular lesion in the synovial lining of the posterior capsule just behind the PCL (Figures 8A,B). An excisional biopsy was performed with a Williams-type discectomy forceps positioned in the posteromedial portal and the arthroscope in the anterolateral portal. We performed partial resection of the surrounding normal synovial lining with a bipolar radiofrequency probe (Dyonics RF Ablation 90° probe, Smith & Nephew, US). Histological exam confirmed arterial and venous proliferation in the synovial stroma, consistent with arteriovenous SH.

Three months after surgery, the patient progressively resumed recreational sports. One year after surgery, the patient was asymptomatic, even when cycling. There was no evidence of recurrence on repeat MRI.

### Literature Review

**Figure 9:**
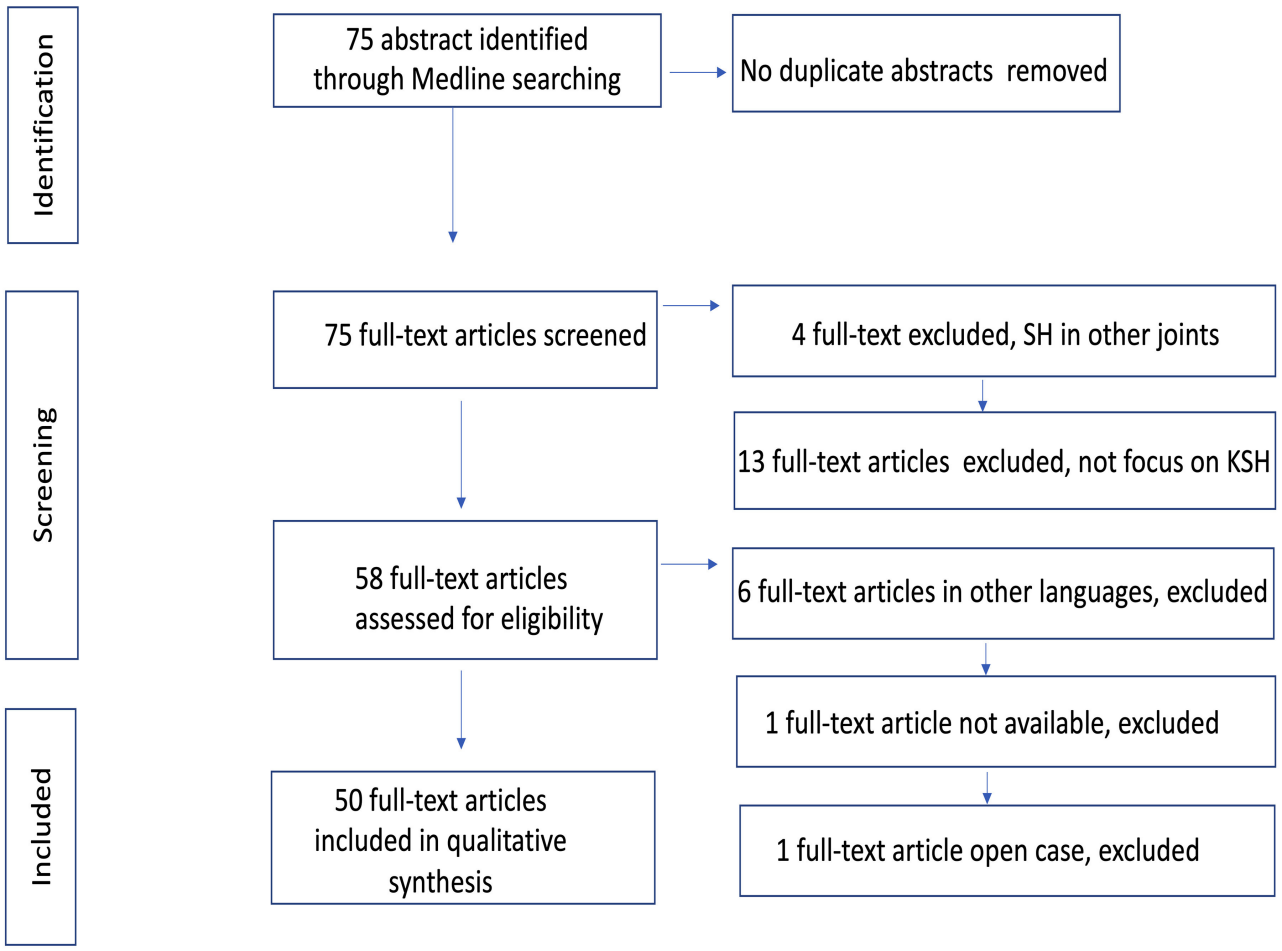
Flow chart of information through the different phases of the systematic review.

We identified 75 abstracts from articles located through the literature search. Eight articles on KSH case descriptions were not reviewed because six were in other languages (three in Chinese, one in Serbian, one in Portuguese and one in Italian), one full-text article from an Indian journal was not available and one was an open radiology case. Four articles described SH in other joints, and 13 did not focus on KSH clinical cases or case series. Ultimately, we reviewed 50 full texts that focused on case or case series descriptions, including a total of 92 clinical cases. A flow chart of the literature search is shown in Figure 9.

The literature review showed that KSH appears more frequently in children and teenagers (64.6% of cases). The average age of presentation was 21.7 ± 14.7 years, and the prevalence was not related to gender (51% males). The right knee was more frequently affected (39.6%).

The tumor mass could appear anywhere in the joint, but an anterior knee location in the suprapatellar and patella-femoral (47.9%) joint space was the most frequent, followed by the infrapatellar (14.6%), posterior (9.4%) and medial compartment (7.3%) and the intercondylar notch (4.2%). Usually, there was no previous trauma (39.6%), or it was not reported (46.9%).

Pain, swelling and haemarthrosis were the most frequently described clinical presentation form (88.2, 66.7, and 47.1%, respectively), although the symptoms and signs associated with KSH were multiple and non-specific (see Table 2). Cutaneous haemangioma was scarce (19.8%) and usually not reported (51%). The diagnosis was normally made late and the average delay was 6.8 ± 8.6 years.

The most commonly reported image tests were plain XR (86.3%) and MRI (98%) alone or enhanced with gadolinium (MRGd). Ultrasound (US), echo Doppler (ED), arteriography, computed tomography (CT) or scintigraphy (Sc) were used less frequently (9.8–5.9%) In rare cases (3.9–2%), venography, CT arthrography (CTArt), CT angiography (CTA), multi-detector computed tomography angiography (MDCTA), red blood cell scintigraphy (RBISc) or single-positron emission computed tomography (SPECT) were reported. Knee bone erosion was occasionally observed (13.5%) on image exams.

Synovectomy was the treatment of choice and was more frequently performed by open surgery (66.7%) than by arthroscopy (15.6%). Treatment options such as observation, pharmacological treatment (corticosteroids and beta blockers), and TKA were rarely recommended (7.3, 1, and 1%, respectively). Biopsy prior to treatment was infrequent (9.4% of cases). The most frequent histologic type was the cavernous type (28.1%), followed by the venous vascular malformation, capillary, arteriovenous and cavernous-capillary types.

The average follow-up time was 2.7 ± 2.2 years, but the follow-up time was unreported in 36% of cases. Reported recurrence was low (3.1%), as it was the persistence of symptoms (11.4%).

## Discussion

Synovial haemangioma have been described in various joints, but the knee is the most common (4). Up to 2015, one paper reported more than 275 cases of KSH (1). Since then, another 24 cases have been reported (PubMed only), with the present four cases bringing the total to 303. There are a number of small case series (4–10, 12, 51–57, 61), the largest describing 14 cases (8). Most reports describe single clinical cases (1–3, 11, 13–50), which highlights the rarity of this type of tumor.

This is the first study that performs a detailed revision of a large number of KSH (96 cases) and describes its more frequent location and clinical presentation forms. The major finding of this study is that plain radiographs and MRI image studies appear to be enough for the differential diagnosis, that will be confirmed after histology exam. Being a tumor mass of benign characteristics on image studies rarely a biopsy is indicated previous to treatment, an excisional biopsy and regional synovectomy can be performed for diagnosis and treatment purposes before diagnostic confirmation. Although open surgery has been the treatment of choice, nowadays arthroscopy seems to offer advantages in the treatment of intraarticular KSH without bone, tendon or muscle involvement. Recurrence after surgery is low (3.1%) but persistence of symptoms is more frequent (11.4%), especially in diffuse forms.

In the last 20 years, only nine cases (9.4%), including ours, had localized lesions in the posterior compartment of the knee. One was an intermediate tumor (5) that extended into the quadriceps and gastrocnemius. Three were juxta-articular, one with an intramuscular component in the popliteal fossa (1) and two behind the posterior cruciate ligament (PCL) (12). The other five were intra-articular, of which three previously reported cases plus our case 4 were behind the PCL (13–16) and one was just cranial to the PCL (17).

Our other three cases were local sessile intra-articular SH located near the superior pole of the patella, by far the most frequent location in the anterior compartment of the knee (5–8, 19, 21, 24, 27–29, 38, 40, 42, 44, 50, 53–57), followed by the infrapatellar fat pad (11, 18, 23, 30, 45, 59), the posterior compartment, the medial compartment (2–4, 25, 35, 39, 42, 54, 61) and the intercondylar notch (22, 43, 46, 47, 54). The three reported patients with KSH near the superior pole of the patella had the rare phenomenon of bone erosion, which was localized in the patella in two cases, a previously undescribed location for KSH bone erosion, and in the supratrochlear cortical bone in one case. It is most frequent in tumors located in the medial compartment (2, 3, 6, 39) or diffusely distributed (31, 34).

KSH usually occurs in children and teenagers (4, 8, 12), although the average age is 21.7 ± 14.7 years. For that reason, the diagnosis is not typically suspected in middle-aged or older adults, as occurred in our patients. The average delay in diagnosis was 6.8 ± 8.6 years, larger than the average noted in one report (6), although two reported patients had a delay in diagnosis of more than 40 years (3, 41).

All four of our patients were male, but we found no differences in sex distribution, and the exclusively male composition of our sample is probably merely a matter of sample size. The right knee is more frequently affected than the left knee. Previous trauma appears to be rare and is often not reported. Symptoms and signs associated with SH of the knee are non-specific. Low-intensity and long-duration pain is the most frequent symptom (4), but it rarely limits activities of daily living except in phases of exacerbation (5, 12, 59). It is usually aggravated with activity and relieved by rest (2, 3, 5, 10, 42, 49). Pain is typically in the anterior aspect of the knee but can be posterior (14) or diffuse. Swelling, tenderness, effusion (5, 9–11, 17, 21, 23, 26, 30, 33, 34, 44, 49, 50, 55) and haemarthrosis (5, 9, 11, 12, 18, 22, 30, 31, 37, 39, 41, 46, 50, 54, 55, 57, 59) are frequent and can be recurrent. Occasionally, a palpable soft tissue mass (3, 4, 10, 44, 46, 55, 61) can be noted. Anterior masses are more evident in complete flexion of the knee (3, 21, 44) and can be compressible, refilling when pressure is released (21). Limitation of motion (3, 5, 8–10, 12, 17, 25, 28, 33, 37, 38, 46, 61) and mild muscular atrophy (5, 11, 33, 34, 36, 49, 55) can be present. Joint locking is infrequent but has been described when synovial haemangioma is located in the intercondylar notch (22, 47) or in the suprapatellar pouch (6). Although up to 40% of patients with synovial haemangioma may have cutaneous haemangioma (4), we appreciate that it is less frequent, probably because patients are often not carefully examined for such haemangiomas, or, if so, they are not reported. Cutaneous haemangioma has been described in patients with diffuse extra-articular KSH (6, 29, 31, 49) or with systemic angiodysplasia (Klippel-Trénaunay) (22, 23).

No clinical test or imaging procedures provide a definitive diagnosis, nor is there a clear algorithm for diagnosis. Plain radiographs are typically normal (4, 8, 11, 13, 16, 17, 21, 27, 28, 35, 38, 39, 41, 44, 47, 49, 50, 53, 55– 57), and only a few cases show non-specific abnormalities (2, 4–10, 33, 36, 45, 53) such as periosteal reaction, osteolysis, osteopenia, intra-articular phleboliths, trochlear dysplasia or osteoarthritis. Even if there is a small region of bone erosion, as in our cases, plain radiographs can appear normal. Formerly, it was the first imaging test ordered because it is fast and inexpensive and permits a general evaluation of the joint, although it is not useful in the diagnosis of KSH.

MRI appears to be the examination of choice (1, 2, 5–11, 16–18, 21–24, 26, 27, 33, 35–40, 42, 43, 46, 48, 50, 53–57). It is non-invasive and non-irradiating and usually allows three-dimensional localization, although occasionally, as in our series, a tumor mass may not be identified (3, 47). However, the features of low or intermediate intensity compared to muscles on T1-weighted sequences and high intensity on T2-weighted or fat-suppressed sequences are not specific to haemangioma (28, 58). Fatty fibrous septa can be seen within the lesion in some cases (57). Large venous haemangiomata can cause high signal intensity on T1 due to slow blood flow (58). Two reports suggested that only 22% of the cases had a correct suspected diagnosis before surgery (2, 10). MRI is useful for demonstrating extension into the periarticular tissues (7), for detecting bone erosion in our cases, for surgical planning, and for following recurrence. Intravenous administration of gadolinium for MRGd produces inhomogeneous enhancement of the synovial tumor image (9, 14, 27, 34, 38, 46, 59) and permits differentiation between cystic synovial hyperplasia (52) and liquid or intra-articular haemangioma (2, 7, 38, 57). MRA and MDCTA have replaced arteriography, and while both are invasive and non-specific methods (20, 50), they may be justified for planning surgery in diffuse or extended cases (3, 32, 46) and in cases where complete excision is not possible and where embolization might be considered (7, 57).

Ultrasound may (19, 23, 24, 46) or may not (8) help distinguish solid vs. liquid masses or those with heterogeneous content. Echo Doppler may suggest a vascular lesion (23, 38, 46, 50, 60), but not always (28). CT is useful in determining the presence and extent of bone involvement (2, 7, 33, 55). Tc99 scintigraphy may show increased uptake in cases where bone is affected (2, 45), RBCSc reveals increased blood pool activity (30), and SPECT/CT shows a radioactive mass (30); however, the information provided is non-specific, so a scan would be justified only in a patient where other imaging examinations are normal.

Diagnostic arthroscopy may be useful in localizing a synovial tumor and ruling out unrelated intra-articular lesions (6, 33, 45, 47). Arthroscopy allows lesion localization; biopsies for frozen section (16, 55), permanent histology or microbiological study; and, in most patients, definitive treatment. We indicate it in the event of persistent knee pain and normal imaging examinations. However, as in case 2, it is not always possible to visualize the tumor (17, 20, 28, 31, 57, 59, 60) or show the complete extent of the lesion (8), either because it is not localized (59) or misdiagnosed (28) or because the lesion is subjacent to the synovial lining and cannot be seen (20).

Histological examination is essential to establish a definitive diagnosis, although it was not reported in 8.3% of reviewed cases. Haemangiomata are tumors derived from endothelial cells, and four types have been described (51). Although three of our cases were of the arteriovenous type, we showed that the most common was the cavernous type, followed by the venous vascular malformation, capillary, arteriovenous and cavernous-capillary types. In 19.8% of the cases, the histological type was not described. It is considered a vascular venous malformation more than a tumor, often associated with a lymphatic hamartomatous component (8).

We advise treating these lesions because they can cause joint destruction either by direct invasion of the surrounding tissues or by repeated haemarthrosis (5, 10, 31). Embolization may be indicated in cases of diffuse lesions, when complete surgical resection is difficult or impossible, or when the tumor mass is nourished by a large blood vessel (7, 36). Non-surgical treatment and observation have been recommended by some authors in diffuse cases (6, 32, 49).

As KSH appears as a synovial tumor mass with benign characteristics in imaging studies, an excisional biopsy and regional synovectomy can be performed until free resection margins are obtained for diagnosis and treatment purposes before diagnostic confirmation. Biopsy prior to treatment is rarely reported (9.4%) but should be indicated if there is doubt regarding the benignity of the tumor based on imaging examinations. There is controversy as to whether open or arthroscopic surgery is better. Most patients have been treated by open surgery because it has been argued that it allows a better view of the full extent of the lesion, mainly when it is located within the Hoffa fat pad, and therefore leads to a lower recurrence rate (4, 8–10, 18, 20, 36, 50, 55). However, recurrence can occur despite complete resection by open surgery (4, 10). Our study found a 3.1% reported recurrence rate with a 11.4% persistence of symptoms rate. We found no large studies reporting recurrence after open vs. arthroscopic resections of localized or diffuse SH, and the reported follow-up times were short or not reported, but PVNS recurrence rates were reportedly comparable between open and arthroscopic surgery (62). We believe open surgery is warranted with juxta-articular or intermediate tumors (50), that is when the tumor extents to the tendons, muscle or bone.

Some authors recommend arthroscopic resection for localized intra-articular KSH (6, 14, 15, 20–22, 33, 36, 38–40, 42, 43, 47, 56, 57, 59, 60). Hoffa fat pad SH can be arthroscopically resected using suprapatellar accessory portals (11, 20, 59). In the past, posterior knee compartment SH was typically treated by open surgery (1, 5, 12, 16, 17) or non-surgically due to predicted difficulty in surgical resection (13); only one case, reported twice, was treated by arthroscopic surgery (14, 15). We performed arthroscopic resection of a posterior compartment SH with resection instruments in the posteromedial and arthroscope in the anterolateral or anteromedial portal (15, 63). This technique avoids possible complications related to an accessory posterolateral portal but has a limited field of view (64). An arthroscopic trans-septal technique using posteromedial and lateral portals may be useful when more working room is needed in the posterior compartment (14, 15, 64). Arthroscopic surgery is advantageous in that it is a minimally invasive technique allowing good assessment of all the joint compartments; it can be used to rule out and treat associated lesions or tumors in the joint; it promotes rapid recovery and superior cosmesis; and it reduces the incidence of postoperative stiffness, infection, thromboembolic events and joint pain (15, 63). An open posterior approach requires a large incision, preventing examination of other parts of the joint, and is associated with relatively poor cosmesis and slow recovery. With the haemostasis achieved by the use of electrocoagulation during arthroscopic tumor resection and synovectomy, open surgery has no advantage in bleeding control (60).

We note several limitations of our study. First, some clinical data were missing in our retrospective clinical review, although key data were available in the records. Second, the literature search was performed only in PubMed and was limited to English, French or Spanish; thus, there is likely additional literature on the subject. However, given the long period over which the papers were published and the large number of cases identified, the included studies are likely representative of the literature. We are unaware of any other such reviews at this level of detail.

It is clinically relevant to know the frequent and unusual locations of KSH, its multiple clinical presentations, the real value of image examinations and the pros and cons of arthroscopic vs. open surgery to be able to make informed decisions on the need for invasive image examinations, previous biopsy or arthroscopic vs. open surgery in light of the evidence.

In conclusion, KSH is a rare tumor. The superior pole of the patella is the most frequent location and only five intra-articular reported cases (including ours) in the posterior knee compartment. SH should be considered in the differential diagnosis of long-term knee pain in adults, particularly if there is no history of previous trauma. Plain radiography is usually normal. MRI is the imaging test of choice to localize a synovial mass, but the images are diagnostically inconclusive. MRA may be useful in diffuse or extended cases. The rare phenomenon of bone erosion, first time described in the patella, have to be bear in mind. Histological examination is necessary for definitive diagnosis. Biopsy prior to treatment should only be indicated if there is doubt regarding the benignity of the tumor. We believe arthroscopic resection should be the surgical technique of choice for treating localized intra-articular lesions or those in difficult areas such as the posterior compartment. We recommend open surgery for diffuse tumors and those that are localized in juxta-articular and intermediate regions with tendon, muscle or bone extension. Complete resection of the synovial lesion and the surrounding affected tissues is important to relieve symptoms and prevent recurrence.

## Data Availability Statement

The raw data supporting the conclusions of this article will be made available by the authors, without undue reservation.

## Author Contributions

JH-H participated in the design of the study, participated in revision of clinical cases and search and revision of bibliography, and interpretation of the data and in drafting the manuscript. JM-B participated in revision of clinical cases and revision of bibliography. EG-O has participated in search and revision of bibliography. FC-S has been involved in search and revision of bibliography. SL-M has participated in search and revision of bibliography, interpretation of data, and drafting the manuscript. All authors contributed to the article and approved the submitted version.

## Conflict of Interest

The authors declare that the research was conducted in the absence of any commercial or financial relationships that could be construed as a potential conflict of interest.

## Publisher's Note

All claims expressed in this article are solely those of the authors and do not necessarily represent those of their affiliated organizations, or those of the publisher, the editors and the reviewers. Any product that may be evaluated in this article, or claim that may be made by its manufacturer, is not guaranteed or endorsed by the publisher.

## Abbreviations

KSH: knee synovial haemangioma
SH: synovial haemangioma
LPVNS: localized pigmented villonodular synovitis
MR: magnetic resonance
MRI: magnetic resonance imaging
MRGd: magnetic resonance enhanced with gadolinium
US: ultrasound
ED: echo Doppler
CT: computed tomography
Sc: scintigraphy
CTArt: computed tomography arthrography
CTA: computed tomography angiography
MDCTA: multi-detector computed tomography angiography
RBCSc: red blood cell scintigraphy
SPECT: single-positron emission computed tomography
TKA: total knee arthroplasty
PCL: posterior cruciate ligament.
